# Supplementary material for: Reconfigurable photoactuator through synergistic use of photochemical and photothermal effects
Source: Nat Commun. 2018 Oct 8;9:4148. doi: 10.1038/s41467-018-06647-7 (PMC6175871; doi:10.1038/s41467-018-06647-7)
Supplement: Supplementary file 1 — Supplementary Information [file 41467_2018_6647_MOESM1_ESM.pdf]

Supplementary Information

Reconfigurable photoactuator through synergistic use of photochemical and photothermal effects

Markus Lahikainen, Hao Zeng*, Arri Priimagi*

Affiliation:

Laboratory of Chemistry and Bioengineering, Tampere University of Technology, P.O. Box 541, FI 33101 Tampere, Finland

*Correspondence to hao.zeng@tut.fi, arri.priimagi@tut.fi

Supplementary Figures

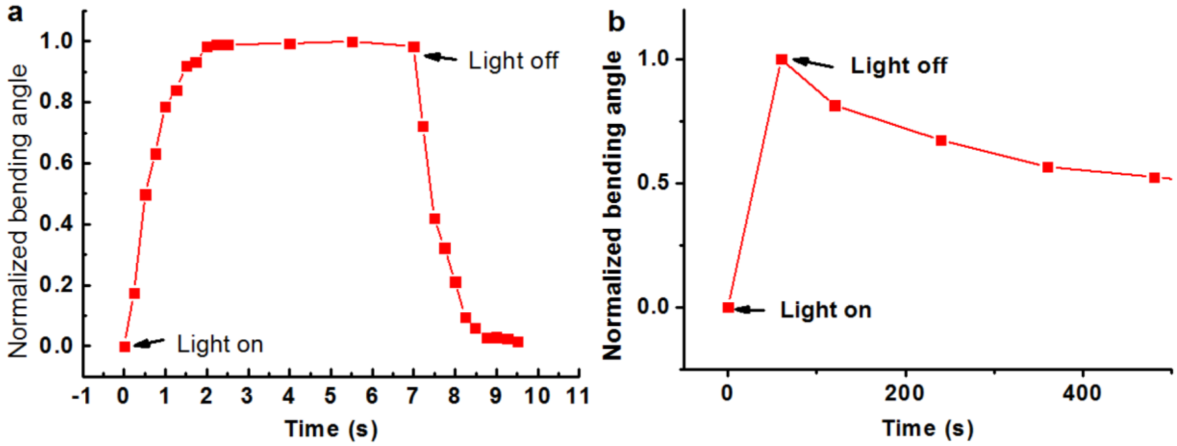

Supplementary Figure 1 |. Actuation dynamics of the LCN stripes. Photothermally (**a**) and photochemically (**b**) induced bending in an LCN strip. Sample: splay-aligned LCN film with 4×1×0.02 mm^3 dimensions. Photochemical actuation is triggered by 365 nm irradiation (9 mW cm^{-2}) and photothermal actuation by 660 nm irradiation (260 mW cm^{-2}).

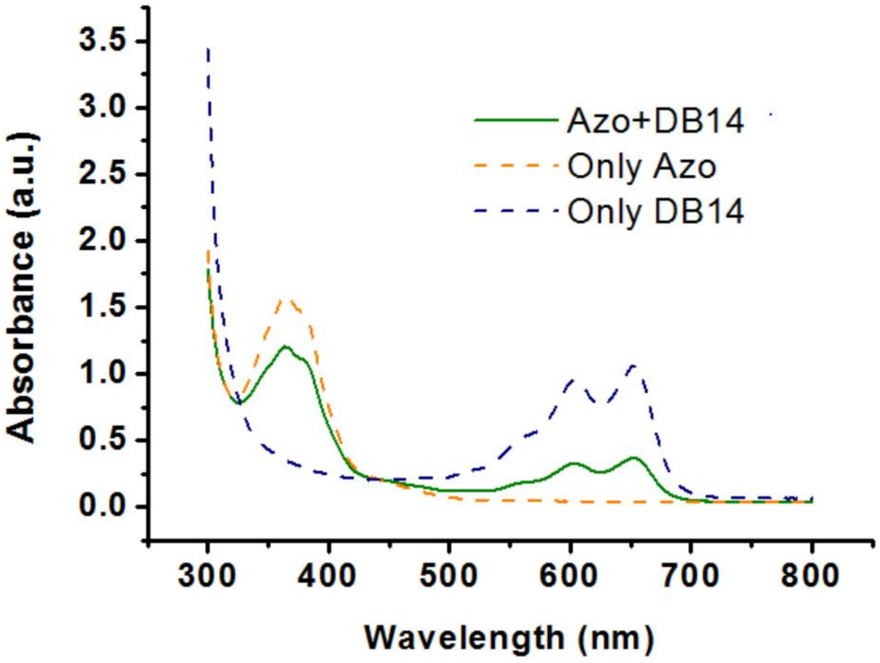

Supplementary Figure 2 |. UV-Vis spectra of LCN films. Films contains only azobenzene crosslinks, only Disperse Blue 14 (DB14), and both (splayed alignment, thickness 10 μm).

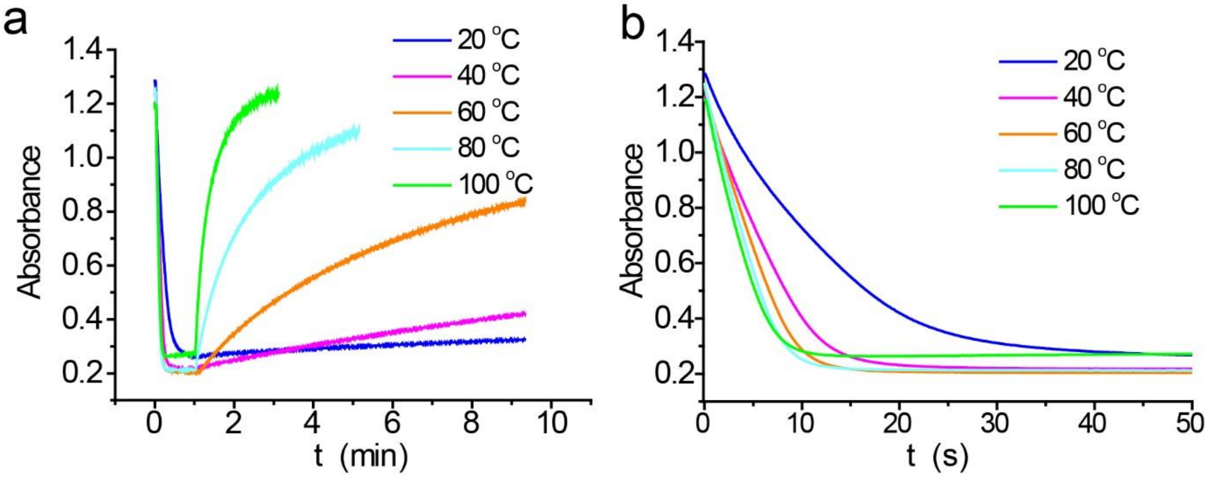

Supplementary Figure 3 |. Isomerization kinetics of the film. a, *Trans-cis* isomerization upon UV exposure and *cis-trans* back relaxation at different elavated temperatures. **b,** Enlarge spectra for *trans-to-cis* isomerization upon UV illumination. UV source: 365 nm LED (18 mW cm^{-2}), switched on at 0 s and ceased at 1 min. Probing wavelength: 385 nm. After each measurement, the sample is exposed to blue light (460 nm, 26 mW cm^{-2}, 1 min) to convert the *cis*-azobenzenes back to the *trans*-form.

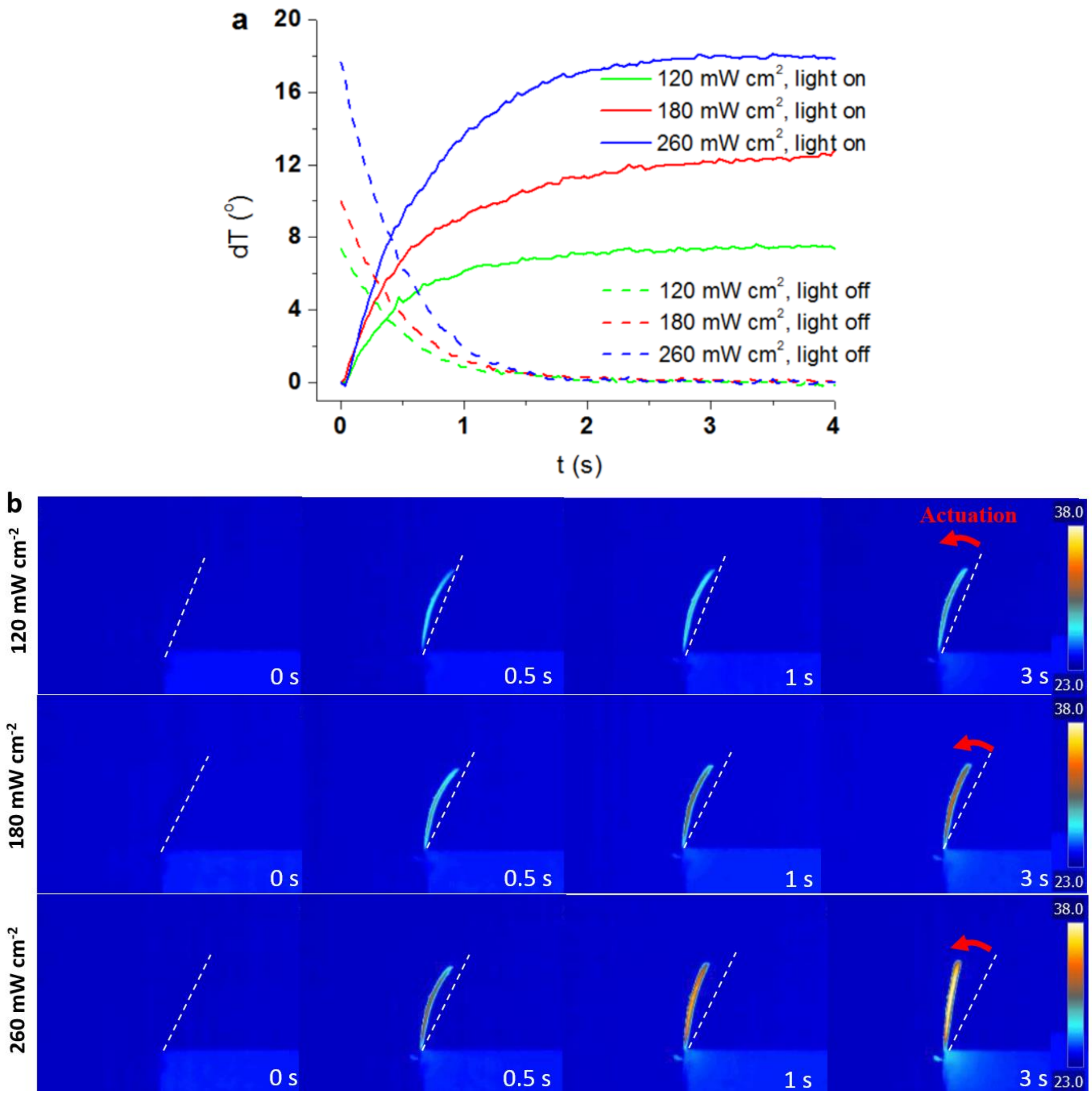

Supplementary Figure 4 |. Photothermal actuation of a splay-aligned LCN strip. **a**, Temperature change induced by different red light illumination intensity. **b**, Series of thermal camera images at different heating stages.

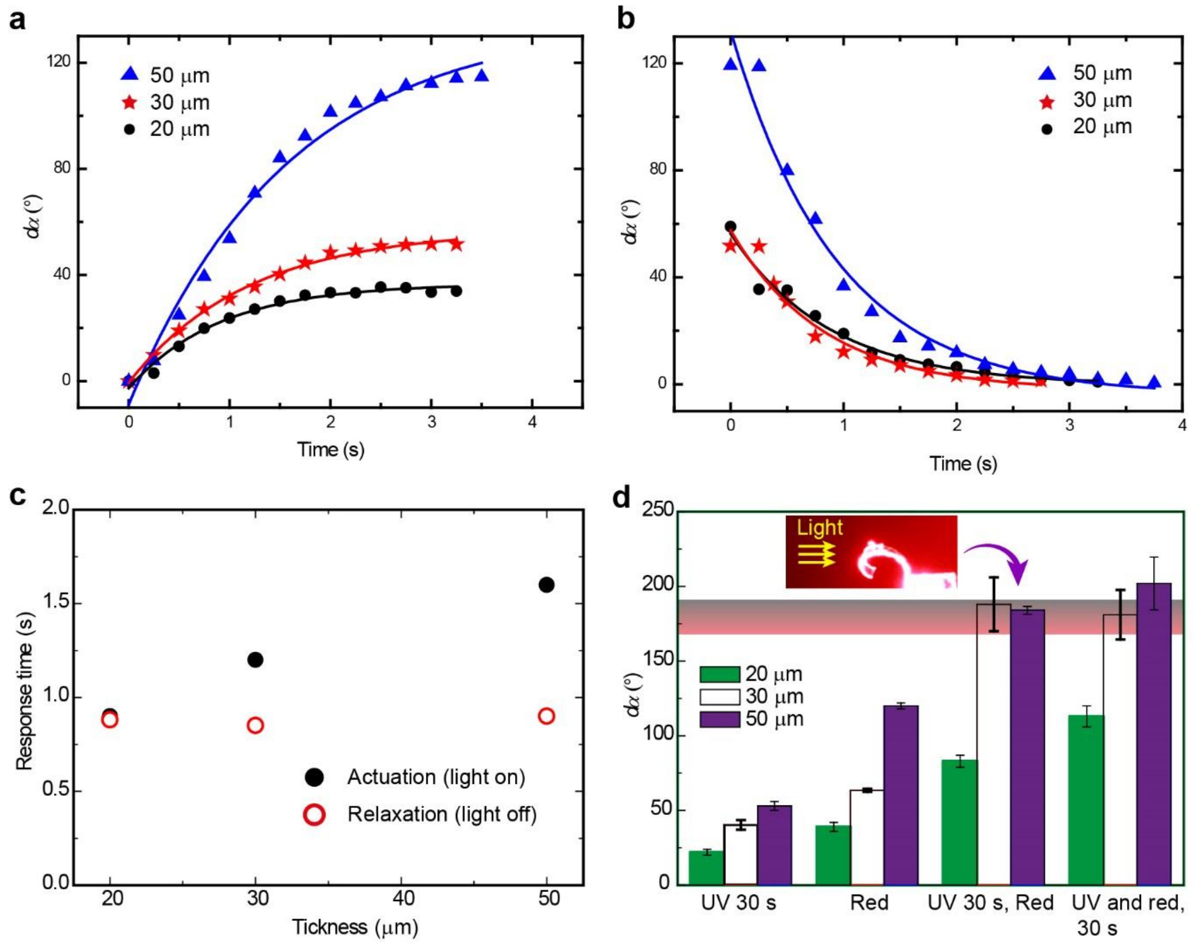

Supplementary Figure 5 |. Photoactuation in LCN strips with different thickness. a, Time response of splay-aligned LCN strips (20, 30, 50 μm thick) upon 660 nm illumination (260mW cm^{-2}). **b**, Relaxation of the same set of LCN strips after ceasing the illumination. Solid lines in **a** and **b** are single exponential fits to the kinetic data. **c**, The response time of actuation/relaxation in strips with different thickness. The reponse time is calculated from the exponetial fitting, determined as deformation reduced to 1/e ≈ 0.37 of its initial value. **d**, Bending angle change upon different stimuli in the strips with different thicknesses. Error bars indicate standard deviation for n = 3 measurements. The bar with color gradient indicates the measurement limitation, above which the deformation starts to saturate due to the light shadowing effect, as shown by the insert photograph. All the strips are 4 mm long and 1 mm in width.

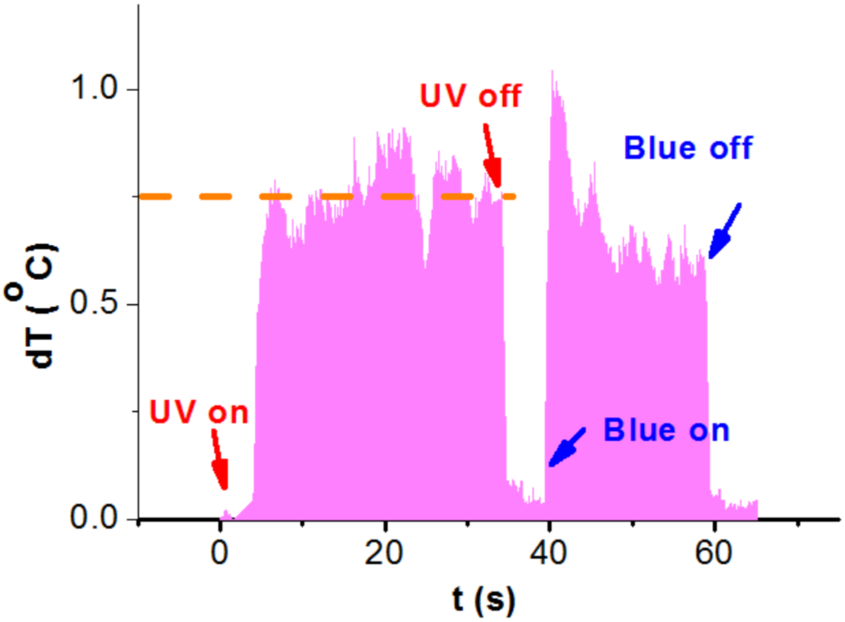

Supplementary Figure 6 |. Temperature increase by photothermal heating. Intensity of the UV light: 9 mW cm^{-2} and blue light: 18 mW cm^{-2} .

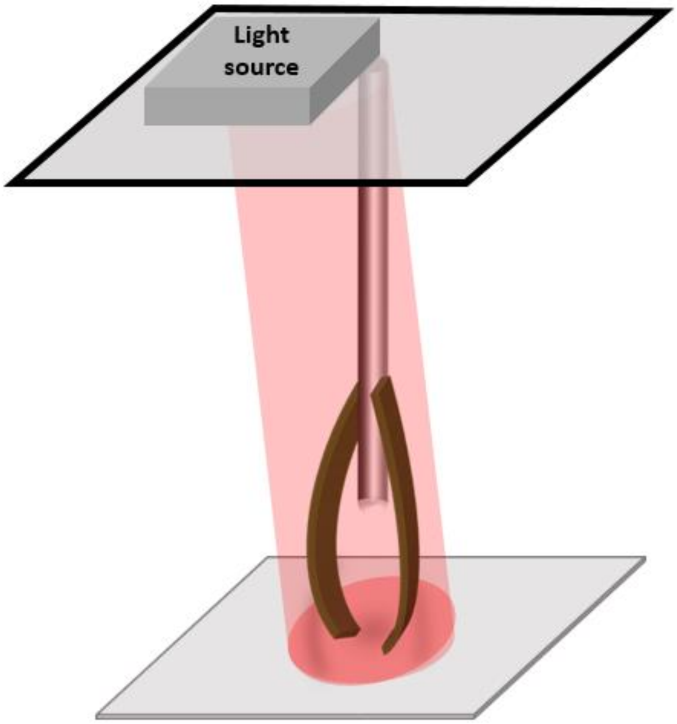

Supplementary Figure 7 |. Optical configuration of light-fuelled gripper.

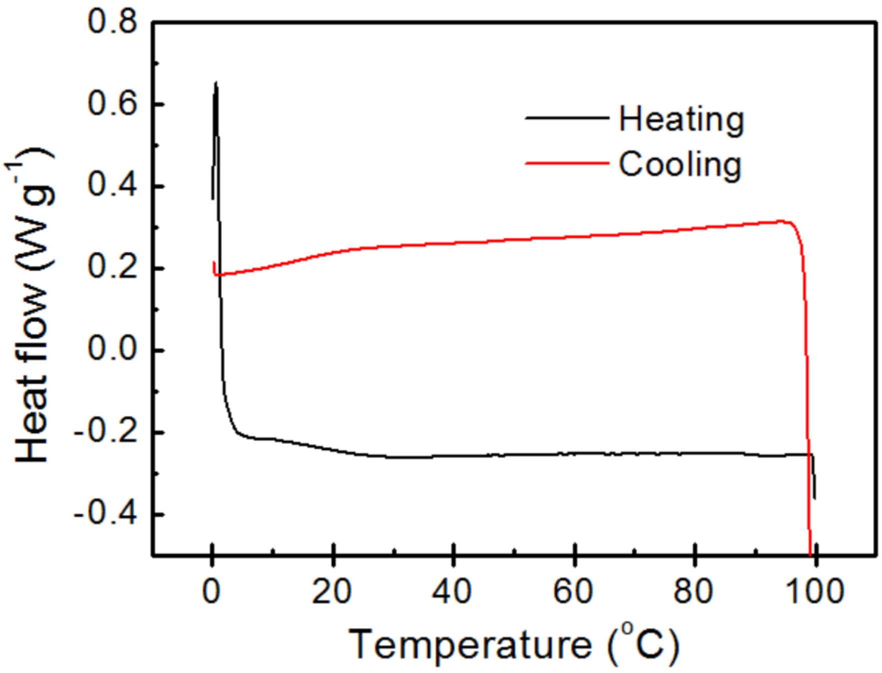

Supplementary Figure 8 |. DSC curves of the LCN film. Heating and cooling speed: 10 °C min^{-1}.

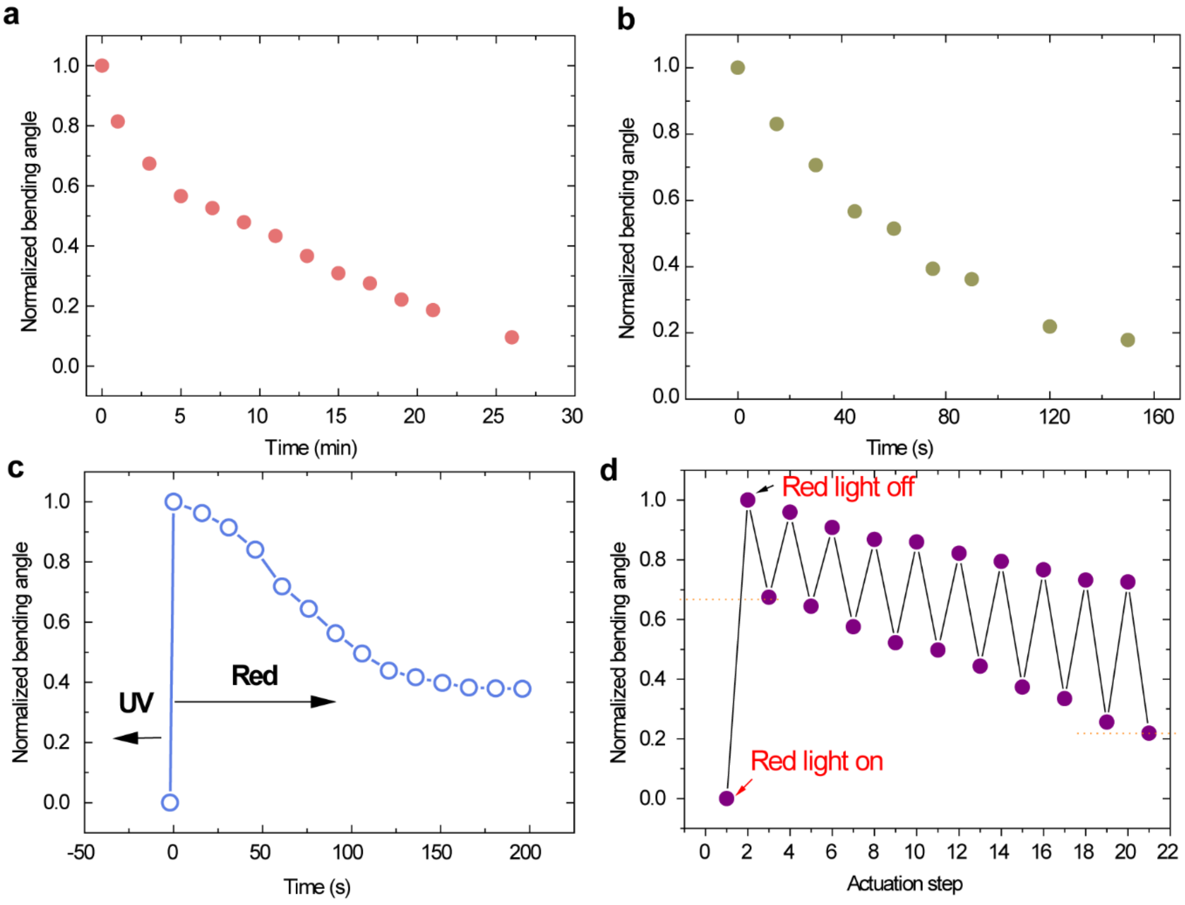

Supplementary Figure 9 |. Thermal relaxation of a bent strip. Relaxation at room temperature (22 °C) under laboratory room light **a**, and under outdoor sunlight, **b**. Sunlight condition: 44.92° altitude angle, (Tampere, N 61°26'55.56", E 23°51'43.88", no cloud); temperature 21 °C. Relaxation upon 260 mW cm^{-2}, 660 nm red light continous excitation **c**, and cyclic exciation **d**. Bending is induced by 365 nm (50 mW cm^{-2}) and 660 nm (260 mW cm^{-2}) illumination for 5 s.

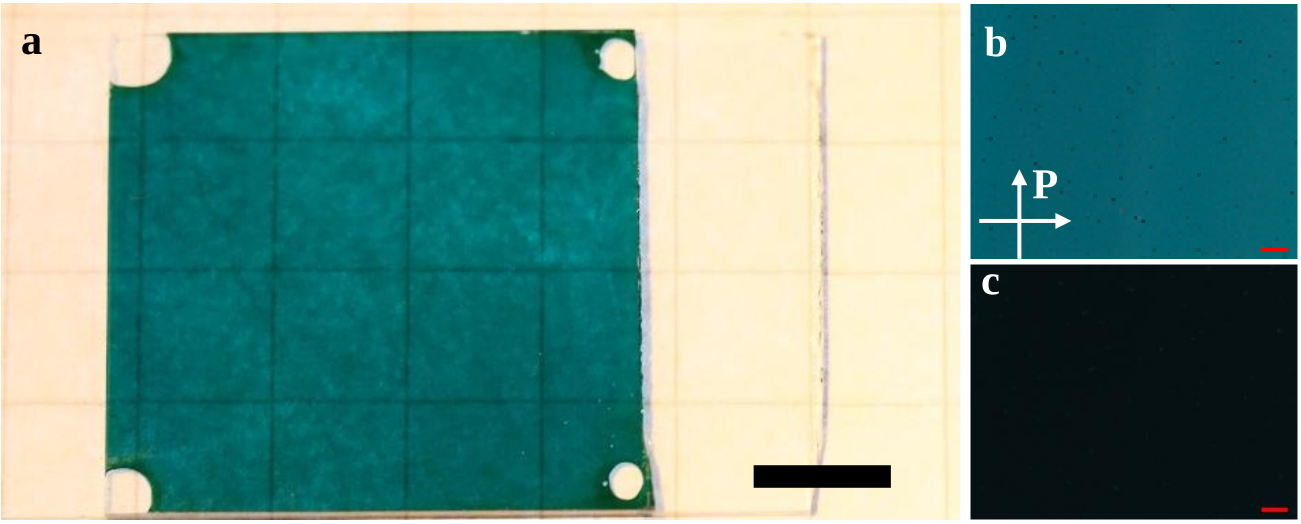

Supplementary Figure 10 |. Imaging of the LCN film. Optical image of a planar-aligned LCN film. Scale bar is 5 mm. **a,** Cross polarized microscopic image of the LCN film placed by matching the alignment direction with one of the polarizer **b**, and by rotating 45° (c). Scale bars: 100 µm.
