# Supplementary material for: Reconfigurable photoactuator through synergistic use of photochemical and photothermal effects
Source: Nat Commun. 2018 Oct 8;9:4148. doi: 10.1038/s41467-018-06647-7 (PMC6175871; doi:10.1038/s41467-018-06647-7)
Supplement: Supplementary file 3 — Description of Additional Supplementary Files [file 41467_2018_6647_MOESM3_ESM.docx]

Description of Additional Supplementary Files

Supplementary Movie 1

Reconfigurable actuation. One single LCN actuator is shape-morphed into six different shapes under red light illumination (660 nm, 300 mW cm^-2^). Schematics indicate the mask used in the pre-UV patterning process (365 nm, 50 mW cm^-2^, 5 sec).

Supplementary Movie 2

Light fueled gripper with Grip-and-drop working model. The device grips an object (mass 12 mg) and holds it in the air when the red light is on (635 nm, 3 W cm^-2^), and dropping it after ceasing the illumination.

Supplementary Movie 3

Light fueled gripper with Grip-and-hold working model. The device grips an object (mass 12 mg) and holds it in the air when the red light is on (635 nm, 3 W cm^-2^), and holding it after ceasing the illumination. The LCN used in this model has been pre-treated under UV illumination (365 nm, 50 mW cm^-2^, 10s).
